# Lifestyle associated factors and risk of urinary bladder cancer: A prospective cohort study from Norway

**DOI:** 10.1002/cam4.3060

**Published:** 2020-04-21

**Authors:** Helga H. Hektoen, Trude E. Robsahm, Bettina K. Andreassen, Jo S. Stenehjem, Karol Axcrona, Alison Mondul, Randi E. Gislefoss

**Affiliations:** ^1^ Department of Research Cancer Registry of Norway Oslo Norway; ^2^ Department of Biostatistics Oslo Centre for Biostatistics and Epidemiology University of Oslo Oslo Norway; ^3^ Department of Urology Akershus University Hospital Lørenskog Norway; ^4^ School of Public Health University of Michigan Ann Arbor MI USA

**Keywords:** body mass index, cohort study, metabolic syndrome, physical activity, urinary bladder cancer

## Abstract

A number of lifestyle associated factors, such as high body mass index (BMI), low physical activity, and related metabolic disorders, are associated with increased risk of cancer at several sites. For urinary bladder cancer (BC), such studies show inconsistent results, which could result from inadequate adjustment for smoking and occupational exposure. In the population‐based Janus Cohort (n = 292 851), we investigated the independent and combined impact of BMI, physical activity, blood pressure, and blood lipids on the risk of BC, by thorough adjustment for smoking and potential occupational exposure. We used cox proportional hazard regression to estimate hazard ratios (HRs) with 95% confidence intervals (CIs) for the associations between the lifestyle associated factors and BC risk. The associations observed were dependent on smoking status and gender. Among men, diastolic blood pressure (DBP) (HR 1.07, 95% CI 1.02‐1.12) and systolic blood pressure (SBP) (HR 1.04, 95% CI 1.01‐1.07) were positively associated with BC risk. Stratification by smoking status revealed a positive association between DBP and BC risk in never smokers (HR 1.14, 95% CI 1.00‐1.30), while no association was seen for current and former smokers. A risk score, integrating information across the lifestyle factors was positively associated with BC risk in men (*p*_trend_ = 0.043). In women, physical activity was associated with a decreased BC risk, but only among never smokers (HR 0.65, 95% CI 0.45‐0.94). In conclusion, relations between lifestyle associated factors and BC risk were most evident in never smokers, suggesting that smoking dominates the relation in current smokers.

## Graphical abstract

In this large population‐based cohort of Norwegian men and women, we have examined relations between lifestyle associated factors (e.g. BMI, physical activity and metabolic disorders) and risk of bladder cancer, with thorough adjustments for smoking and occupational exposure. We found that relations between lifestyle associated factors and BC risk were dependent on smoking status, being most evident in never and former smokers, suggesting that smoking dominates the relation in current smokers.

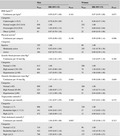

**Abbreviations**
BC: urinary bladder cancer
BMI: body mass index;
CI: confidence interval
DBP: Diastolic blood pressure
HR: hazard ratio
Me‐Can: The metabolic syndrome and cancer study
SBP: Systolic blood pressure
SD: standard deviation

## 1 INTRODUCTION

Urinary bladder cancer (BC) is the 10th most common cancer worldwide, with nearly 550,000 new cases in 2018.^1^ The incidence rates are highest in developed countries, where urothelial BC is the predominant histologic type, and the rates are 3‐4 times higher in men compared to women.^2^, ^3^, ^4^ In Norway, BC is the 4th most common cancer in men.^5^ The most important risk factors are smoking, accounting for up to 50% of the cases,^6^ and occupational exposures (eg aromatic amines and polycyclic hydrocarbons).^7^, ^8^, ^9^, ^10^ As smoking rates are declining in many parts of the world and occupational hygiene has improved, the incidence of BC has decreased in some European countries.^11^, ^12^, ^13^ However, in white Americans the incidence rates have remained stable,^8^ and in Norway the rates are still increasing,^5^ suggesting that other risk factors also could play a role in BC etiology.

A number of lifestyle associated factors, such as high body mass index (BMI), low physical activity, and related metabolic disorders have been found to increase the risk of cancer at several sites,^14^, ^15^, ^16^, ^17^ whereas BC studies have shown conflicting results.^18^, ^19^, ^20^, ^21^ However, a meta‐analysis examining the association between BMI and BC found that a BMI > 25 kg/m^2^ increased the risk.^22^ Likewise for physical activity, a meta‐analysis has shown a 15% decreased risk of BC associated with high levels of physical activity compared to lower levels.^23^ Metabolic disorders, such as hypertension and dyslipidemia, are associated with both high BMI and low levels of physical activity,^24^, ^25^ and emerging evidence suggests that a metabolic profile associated with obesity may be a more relevant risk factor for some cancers than obesity alone.^26^ A large meta‐analysis has shown associations between metabolic disorders and cancer risk at several sites, including BC in men.^17^ Hypertension, as an independent metabolic factor has also been shown to increase the risk.^27^, ^28^ However, the literature regarding metabolic disorders and BC risk is inconsistent.^29^

The inconclusive results seen in the association between BC risk and BMI, physical activity, and metabolic disorders, might be a consequence of these variables’ being related to peoples’ smoking habits,^30^, ^31^, ^32^ the most dominant risk factor of BC. Although, sufficient adjustment for smoking is crucial, few studies to date have had detailed information on smoking intensity and duration to conduct analysis incorporating adequate adjustment for smoking when reporting on a population based level.^22^ In addition, few studies have had sufficient number of individuals to be able to conduct analyses stratified by smoking status, which has been highlighted as important in recent studies.^19^, ^22^

The metabolic syndrome and cancer study (Me‐Can), a large pooled cohort comprising cohorts from Norway, Sweden, and Austria, found associations between metabolic factors and BC risk after adjustment for pack years.^33^, ^34^ The current study population represents the Norwegian sub‐population of the Me‐Can study. However, as we solely focused on the Norwegian population, we had the possibility to include additional exposures like the potential confounder occupation, and take a closer look at the role of smoking in the relation between metabolic factors and bladder cancer risk.

In this large population‐based cohort of Norwegian men and women, we aimed to investigate the independent and combined impact of; BMI, physical activity, hypertension, and dyslipidaemia on the risk of BC, when thoroughly adjusting for smoking and potential exposure from occupation.

## 2 MATERIALS AND METHODS

### 2.1 Study subjects and data collection

The Janus Serum Bank Cohort (Janus Cohort) has been created as a population‐based biobank for prospective cancer studies, containing serum samples and data from health examinations, including measured anthropometry and questionnaire data from 292,851 Norwegians who participated in at least one of the following five regional health studies conducted in Norway between 1972 and 2003; the Oslo study I (1972‐1973), the Norwegian Counties Study (1974‐1978, 1977‐1983 and 1985‐1988), the Oslo Age 40 Program (1981‐1999), the National Age 40 Program (1985‐1999), and the Tromsø and Finnmark Health Study (2001‐2003). Detailed description of the samples and data included in the Janus Cohort has been published elsewhere.^35^, ^36^

The present study is based on an analytic dataset created through linkages between the health information (measured anthropometry and blood lipids) and questionnaire data from the Janus Cohort, and individual information on education, occupation, cancer diagnosis, vital status, date of death, and emigration from national registries. Details of the data sources and the linkages are available in the published study protocol.^37^ Approvals for the data linkages and for conducting the study were obtained from the Regional Committee for Medical Research Ethics.

### 2.2 Outcome, study population, and follow‐up

BC cases were identified by linkage with the Cancer Registry of Norway, which has registered cancer diagnoses since 1953, by law, and holds complete data of high quality.^38^ The outcome of interest in this study was BC risk. We identified all BC cases diagnosed between 1972 and 2016, without any cancer history. We only included BC cases of the transitional cell type, defined by using International classification of disease for Oncology 3rd revision (morphological codes: 8120, 8130, and 8131). The information about tumor invasiveness was based on pathological histology reports. Among the 1,978 BC cases included, 1,584 were categorized as nonmuscle invasive bladder cancer (NMIBC), including papillary noninvasive tumors (Ta), carcinoma in situ (Tis) and tumors invading lamina propria (T1), and 394 were categorized as muscle invasive bladder cancer (MIBC), including tumors invading muscularis propria and further (T‐stage T2‐T4).

From the Janus Cohort (n = 292,851), we excluded 76 individuals with a BC diagnosis before baseline, and seven who had died or emigrated before baseline, leaving 292,768 individuals available for analysis. Study entry was defined as the year each individual first participated in the Janus Cohort between 1972 and 2003. Subjects were followed from study entry until BC diagnosis, death, emigration, or until end of follow‐up (December 31, 2016), whichever occurred first.

### 2.3 Exposure assessment

At study entry (between 1972 and 2003), data from health examination and questionnaire data was collected from each individual.

Information on smoking was self‐reported and abstracted from the questionnaires. The questions about smoking status were worded differently between the surveys, and were therefore harmonized into the following categories: current, former, and never smoker.^35^ Pack years were estimated by multiplying number of packs (cigarettes smoked per day/20) with number of years smoked,^39^ and categorized in quintiles. The number of cigarettes were either reported as categorical or continuous, when categorical, the median of each category was assigned to create a continuous variable.

Measurements of height (measured to the nearest cm) and weight (measured to the nearest 0.5 kg), were performed by trained staff according to a standard protocol. BMI was calculated as kg/m^2^, and categorized according to the World Health Organization's classification: underweight (<18.5 kg/m^2^), normal weight (18.5‐24.9 kg/m^2^) overweight (25‐29.9 kg/m^2^), and obese (≥30 kg/m^2^).

Leisure time physical activity was assessed according to the questionnaires used in the health surveys, which previously have been validated against various measures.^40^ The participants were asked about their leisure time physical activity on a regular week over the last year. The responses were categorized into the following three categories: (1) Inactive: reading, watching TV or other sedentary activities, (2) Moderately active: walking, bicycling ≤ 4 hours per week, (3) Active: light sport, heavy gardening ≥ 4 hours per week and/or hard exercise, competitive sports regularly.^35^

Blood pressure was measured in sitting position, after a minimum of 2 min rest, using a manual mercury sphygmomanometer (until the late 1980s) or an automatic device (from the late 1980s onward). These methods have been found to be comparable.^41^ Two or three repeated measurements were assessed, depending on the cohort. If two measurements were recorded, the second measurement was used, and if three measurements were recorded, the mean value of the second and third measurement was used.^42^, ^43^ Systolic blood pressure (SBP) was categorized as normal (SBP < 130), high normal (SBP 130 ‐ 139), and hypertension (SBP ≥ 140) and diastolic blood pressure was categorized as normal (DBP < 85), high normal (DBP 85‐89), and hypertension (DBP ≥ 90), according to the 2018 European Society of Cardiology/European Society of Hypertension (ESH/ECH) Clinical Practice Guidelines.^44^ Information about blood pressure medication was based on self‐reported information, as the participants were asked if they were under treatment for hypertension.

Concentrations of total cholesterol and triglycerides were measured in nonfasting blood samples, using a nonenzymatic method (until 1979) and thereafter an enzymatic method. Serum values from the nonenzymatic method have been transformed by a calibration equation to correspond to the enzymatic method.^45^ The levels of total cholesterol were categorized as normal (<5.2 mmol/L), moderately high (5.2‐6.1 mmol/L), and high (6.2 mmol/L) and triglyceride levels were categorized as normal (<1.7 mmol/L), moderately high (1.7‐2.2 mmol/L), and high (>2.3 mmol/L), both according to cut points from the US National Cholesterol Education Program.^46^

Occupational information was obtained from Norwegian census records from 1970 and 1980. Based on current knowledge of occupational exposures related to BC risk,^47^, ^48^ each occupational working title was scored dichotomously, 1 or 0, depending of being in the category as a high‐risk occupation or not. A detailed list of high‐risk occupations is presented as supplementary material (Supplementary Table S1). Information about educational level was categorized as none, compulsory, upper secondary, college/university, and unknown.

### 2.4 Lifestyle associated risk score

We generated a lifestyle associated risk score based on the following variables; BMI, physical activity, blood pressure (DBP and SBP), total cholesterol, and triglycerides. The variables were classified based on current national guidelines defining threshold of critical levels associated with health risk.^15^, ^44^, ^46^, ^49^ At risk was defined as: 1. BMI > 25 kg/m2, 2. Physical inactive, 3. Hypertension (SBP > 140 mmHg, and/or DBP > 90 mmHg), 4. Triglyceride levels > 2.3 mmol/L, 5. Total cholesterol > 6.2 mmol/L. Each factor was scored dichotomously, 1 if at risk and 0 if not. A combined score was created by summing over each factor, where 0 was the lowest possible score and 5 was the highest.

### 2.5 Statistical analysis

Cox proportional hazards regression was used to estimate hazard ratios (HRs) with 95% confidence intervals (CIs) for the associations between the lifestyle associated factors and risk of BC. Age was used as the underlying time scale. Individuals were followed from the age they entered the study and until censoring (age at diagnosis of BC, emigration, death, or end of follow‐up, whichever occurred first). The multivariable models were adjusted for attained age (as the time scale), BMI, physical activity, smoking status in seven categories (never smoker, former smoker, and current smoker in five categories of pack years), occupation, and education.

Tests for linear trend across categories were performed by analyzing the continuous variable, except for physical activity for which the ordinal variable was entered as continuous in the regression model. Compliance with the proportional hazard assumption was tested by evaluating Schoenfeld residuals and log‐log plots for all covariates, stratified by sex, and indicated no violation of the assumption. To adjust for cohort effects, we included cohort as a variable in the model.

All statistical analyses were performed using the statistical software package version 15.1 (StataCorp, College Station, TX, USA). The significance level was set to 5%, and all tests were two‐sided.

## 3 RESULTS

The study included 152,505 (52%) men and 140,263 (48%) women. Baseline characteristics stratified by gender are presented in Table 1. The mean age at baseline was 42 years (both genders) and the mean follow‐up time was 29 years, ranging from 1 to 45 years. During follow‐up, 1978 BC cases were diagnosed (1619 in men and 359 in women). A larger proportion of current smokers was seen in men (45%) than in women (40%) and a larger proportion of never smokers was seen in women (40%) than in men (26%). Moreover, a larger proportion of the men were overweight or obese (48%) than women (32%). Almost a three folded proportion of the men were in the highest category of physical activity (28%), compared to women (10%). Women had lower blood pressure (both SBP and DBP), levels of triglyceride and cholesterol, compared to men.

**TABLE 1:** Characteristics of study population, by sex

Characteristics	Men	Women
Participants, n (%)	152,505 (52)	140,263 (48)
Year of birth (range)	1942 (1900‐1976)	1944 (1900‐1976)
Age at baseline, mean years (range)	42 (15‐89)	42 (15‐89)
Years of follow up (range)	28 (1‐45)	29 (1‐43)
Bladder cancer cases (n, % of cases)	1619 (82)	359 (18)
Age at cancer diagnosis	66 (39‐91)	64 (38‐89)
*Smoking habits*		
Smoking status, n (%)		
Never	40,320 (26)	55,470 (40)
Former	40,519 (27)	25,393 (18)
Current	68,737 (45)	55,699 (40)
Packyears of smoking, mean (SD)	15.2 (9.4)	11.1 (7.8)
*BMI, kg/m^2^*		
Mean (SD)	25.2 (3.1)	24.2 (4.0)
Category, n (%)		
Underweight (<18.5)	690 (0.5)	2869 (2)
Normal weight (18.5‐24.9)	78,298 (51)	91,273 (65)
Overweight (25.0‐29.9)	62,653 (41)	34,135 (24)
Obese (>30.0)	10,488 (7)	11,644 (8)
*Physical activity*		
Category, n (%)		
Sedentary	29,790 (20)	28,871 (21)
Moderately active	79,780 (52)	95,746 (68)
Active	42,017 (28)	14,641 (10)
*Sytosolic bloodpressure, mmHg*		
Mean (SD)	136 (16)	128 (17)
Category, n (%)		
Normal (<130)	55,542 (36)	85,155 (61)
High normal (130‐139)	41,812 (27)	26,666 (19)
High (≥140)	55,019 (36)	28,377 (20)
*Diastolic bloodpressure, mm Hg*		
Mean (SD)	83 (11)	78 (11)
Category, n (%)		
Normal (<85)	90,047 (59)	104,655 (75)
High Normal (85‐89)	22,417 (15)	15,092 (11)
High (≥90)	39,906 (26)	20,453 (15)
*Triglycerides, mmol/L*		
Mean (SD)	2.0 (1.3)	1.3 (0.8)
Category, n (%)		
Normal (<1.7)	74,380 (49)	110,336 (79)
Borderline high (1.7‐2.2)	32,577 (21)	16,636 (12)
High (≥2.3)	45,512 (30)	13,271 (9)
*Cholesterol, mean mmol/L*		
Mean (SD)	6.0 (1.2)	5.7 (1.1)
Category, n (%)		
Normal (<5.2)	35,729 (23)	46,439 (33)
Borderline high (5.2‐6.1)	54,751 (36)	52,417 (37)
High (≥6.2)	62,007 (41)	41,387 (30)
*High risk occupation, no/yes, n (%)*		
No	101,798 (67)	109,526 (78)
Yes	46,754 (31)	5,463 (4)
*Education, n (%)*		
None	512 (0.3)	461 (0.3)
Compulsory	45,965 (30)	46,982 (34)
Upper secondary	74,713 (50)	69,928 (50)
College/university	30,711 (20)	22,433 (16)

Baseline characteristics varied by smoking status (Supplementary Table S2 and S3). In men, a lower proportion were overweight or obese among current smokers (44%) compared to never (48%) and former smokers (55%). Similar for women, a lower proportion were overweight or obese among current smokers (28%), compared to never (37%) and former smokers (35%). Furthermore, both men and women that were current smokers showed patterns of being less physically active, having higher cholesterol and triglyceride levels compared to never smokers.

In men, we observed no association between BMI and the risk of BC. Physical activity was inversely associated to BC risk, with a lower HR (0.87, 95% CI 0.75‐1.01) for the active compared to the inactive category, although not statistically significant (Table 2) The risk of BC was found to increase with increasing levels of both SBP (HR 1.04, 95% CI 1.01‐1.07) and DBP (HR 1.07, 95% CI 1.02‐1.12). Compared to normal levels, increased risk was found for elevated DBP (≥90mmHg) (HR 1.21, 95% CI 1.08‐1.36). No associations were found for triglycerides and cholesterol. In women, no associations with BC risk were found for any of the lifestyle associated factors studied. In men, stratification by smoking revealed a higher risk of BC (HR 1.24, 95% CI 1.01‐1.52) in former smokers with overweight (BMI 25‐29.9 kg/m^2^), compared to former smokers with normal weight (Table 3). In addition, we found a stronger association between DBP and BC in never smokers, with significantly elevated HR (1.57, 95% CI 1.15‐2.14) for DBP hypertension (≥90mmHg), compared to normal DBP levels. No associations were found for physical activity, triglycerides, or cholesterol, in any strata of smoking status.

**TABLE 2:** Hazard ratio (HR) with 95% confidence interval (CI) of bladder cancer according to body mass index (BMI), physical activity, systolic blood pressure, diastolic blood pressure, triglycerides, and total cholesterol

	Men			Women		
	n_cases_	HR (95% CI)	*p_trend_*	n_cases_	HR (95% CI)	*p_trend_*
BMI (kg/m^2^)^a^						
Continuous per kg/m^2^		0.99 (0.97‐1.00)	0.161		0.97 (0.95‐1.00)	0.071
Categories						
Underweight (<18.5)	5	0.70 (0.29‐1.69)		8	0.96 (0.47‐1.94)	
Normal weight (18.5‐24.9)	858	1.00		252	1.00	
Overweight (25.0‐29.9)	666	1.02 (0.92‐1.13)		77	0.80 (0.62‐1.04)	
Obese (≥30.0)	83	0.87 (0.70‐1.10)		22	0.69 (0.45‐1.08)	
Physical activity^b^						
Continuous per category		0.95 (0.90‐1.02)	0.148		0.99 (0.90‐1.14)	0.906
Categories						
Inactive	355	1.00		80	1.00	
Moderately active	874	0.92 (0.81‐1.04)		245	1.01 (0.78‐1.30)	
Active	384	0.87 (0.75‐1.01)		32	0.89 (0.59‐1.34)	
Systolic blood pressure (mm Hg)^c^						
Continuous per 10 mm Hg		1.04 (1.01‐1.07)	0.018		1.03 (0.97‐1.10)	0.305
Categories						
Normal (<130)	613	1.00		206	1.00	
High normal (130‐139)	404	0.91 (0.80‐1.04)		78	1.20 (0.92‐1.56)	
Hypertension (≥140)	601	1.07 (0.95‐1.20)		75	1.06 (0.80‐1.40)	
Diastolic bloodpressure (mm Hg)^c^						
Continuous per 10 mm Hg		1.07 (1.02‐1.12)	0.004		0.98 (0.88‐1.08)	0.669
Categories						
Normal (<85)	890	1.00		268	1.00	
High Normal (85‐89)	223	1.00 (0.87‐1.17)		40	1.03 (0.73‐1.43)	
Hypertension (≥90)	505	1.21 (1.08‐1.36)		51	0.94 (0.69‐1.28)	
Triglycerides (mmol/L)^c^						
Continuous per mmol/L		1.01 (0.97‐1.05)	0.589		0.93 (0.81‐1.08)	0.372
Categories						
Normal (<1.7)	806	1.00		320	1.00	
Borderline high (1.7‐2.3)	341	0.94 (0.83‐1.07)		27	1.12 (0.82‐1.52)	
High (≥2.3)	472	1.00 (0.89‐1.13)		12	0.90 (0.61‐1.33)	
Total cholesterol (mmol/L)^c^						
Continuous per mmol/L		1.04 (0.99‐1.08)	0.093		1.05 (0.96‐1.15)	0.313
Categories						
Normal (<5.2)	310	1.00		96	1.00	
Borderline high (5.2‐6.1)	522	0.95 (0.83‐1.10)		121	1.02 (0.78‐1.33)	
High (≥6.2)	768	1.05 (0.91‐1.20)		137	1.19 (0.90‐1.57)	

Note

Cox proportional hazard regression models were adjusted for:

^a^ Adjusted for age as the underlying time scale, physical activity, smoking status and packyears, education and high risk occupation.

^b^ Adjusted for age as the underlying time scale, BMI, smoking status and packyears, education and high risk occupation.

^c^ Adjusted for age as the underlying time scale, BMI, physical activity, smoking status and packyears, education and high risk occupation.

**TABLE 3:** Hazard ratio (HR) with 95% confidence interval (CI) of bladder cancer risk among** men** according to body mass index (BMI), physical activity, systolic blood pressure, diastolic blood pressure, triglycerides and total cholesterol, stratified by smoking status

	Never smokers			Former smokers^d^			Current smokers^d^		
	n_cases_	HR (95% CI)	*p_trend_*	n_cases_	HR (95% CI)	*p_trend_*	n_cases_	HR (95% CI)	*p_trend_*
BMI (kg/m^2^)^a^									
Continuous per kg/m^2^		0.99 (0.95‐1.04)	0.815		0.99 (0.96‐1.03)	0.655		0.98 (0.96‐1.01)	0.157
Categories									
Underweight (<18.5)	0	‐		1	1.40 (0.20‐10)		4	0.69 (0.26‐1.8)	
Normal weight (18.5‐24.9)	107	1.00		165	1.00		574	1.00	
Overweight (25.0‐29.9)	84	0.96 (0.72‐1.29)		210	1.24 (1.01‐1.52)		367	0.95 (0.83‐1.1)	
Obese (≥30.0)	10	0.77 (0.40‐1‐49)		18	0.69 (0.42‐1.13)		55	0.99 (0.75‐1.3)	
Physical activity^b^									
Continuous per category		0.89 (0.74‐1.06)	0.182		1.03 (0.92‐1.16)	0.568		0.94 (0.87‐1.03)	0.176
Categories									
Sedentary	30	1.00		65	1.00		255	1.00	
Moderately active	114	1.10 (0.73‐1.64)		215	0.94 (0.71‐1.24)		539	0.90 (0.79‐1.05)	
Active	56	0.80 (0.51‐1.25)		112	0.98 (0.72‐1.34)		210	0.88 (0.73 −1.06)	
Systolic blood pressure (mm Hg)^c^									
Continuous per 10 mm Hg		1.02 (0.93.1.12)	0.705		1.05 (0.99‐1.11)	0.110		1.03 (0.99‐1.07)	0.184
Categories									
Normal (<130)	72	1.00		137	1.00		393	1.00	
High normal (130‐139)	47	0.85 (0.59‐1.24)		94	0.86 (0.66‐1.13)		259	0.93 (0.79‐1.09)	
Hypertension (≥140)	81	1.09 (0.79‐1.52)		164	1.05 (0.83‐1.33)		354	1.04 (0.90‐1.20)	
Diastolic bloodpressure (mm Hg)^c^									
Continuous per 10 mm Hg		1.14 (1.00‐1.30)	0.058		1.07 (0.97‐1.17)	0.177		1.05 (0.99‐1.12)	0.084
Categories									
Normal (<85)	98	1.00		196	1.00		586	1.00	
High normal (85‐89)	26	1.03 (0.67‐1.59)		58	1.06 (0.79‐1.42)		134	0.95 (0.79‐1.15)	
Hypertension (≥90)	76	1.57 (1.15‐2.14)		141	1.19 (0.95‐1.49)		286	1.14 (0.99‐1.32)	
Triglycerides (mmol/L)^c^									
Continuous per mmol/L		0.93 (0.81‐1.07)	0.313		1.04 (0.96‐1.13)	0.328		1.01 (0.96‐1.06)	0.686
Categories									
Normal (<1.7)	117	1.00		200	1.00		477	1.00	
Borderline high (1.7‐2.2)	34	0.74 (0.51‐1.09)		70	0.80 (0.61‐1.06)		234	1.03 (0.88‐1.20)	
High (≥2.3)	50	0.87 (0.61‐1.24)		125	1.13 (0.89‐1.43)		295	0.97 (0.83‐1.13)	
Total cholesterol (mmol/L)^c^									
Continuous per mmol/L		1.04 (0.92‐1.18)	0.531		1.01 (0.93‐1.10)	0.818		1.04 (0.98‐1.09)	0.178
Categories									
Normal (<5.2)	54	1.00		72	1.00		181	1.00	
Borderline high (5.2‐6.1)	62	0.79 (0.54‐1.13)		144	1.12 (0.84‐1.49)		307	0.91 (0.75‐1.09)	
High (≥6.2)	81	1.00 (0.70‐1.43)		176	1.05 (0.80‐1.38)		506	1.02 (0.86‐1.21)	

Note

Cox proportional hazard regression models were adjusted for:

^a^ Adjusted for age as the underlying time scale, physical activity, education, and high risk occupation.

^b^ Adjusted for age as the underlying time scale, BMI, education, and high risk occupation.

^c^ Adjusted for age as the underlying time scale, BMI, physical activity, education, and high risk occupation

^d^ Former and current smokers are additionally adjusted for pack years.

In women, we observed a decreased risk of BC (HR 0.26, 95% CI 0.09‐0.73) in former smokers with overweight, compared to former smokers with normal weight, and in obese current smokers (HR 0.46 95% CI 0.22‐0.99) compared to current smokers with normal weight (Table 4). In never smoking women, reduced risks were found for the moderate (HR 0.63, 95% CI 0.39‐1.00) and the most physically active (HR 0.47, 95%CI 0.20‐1.08) compared to the sedentary, although the latter was not statistically significant. No significant associations were observed between blood pressure, triglycerides, or cholesterol and BC, in any strata of smoking status.

**TABLE 4:** Hazard ratio (HR) with 95% confidence interval (CI) of bladder cancer risk among women according to body mass index (BMI), physical activity, systolic blood pressure, diastolic blood pressure, triglycerides and total cholesterol, stratified by smoking status

	Never smokers			Former smokers^d^			Current smokers^d^		
	n_cases_	HR (95% CI)	*p _trend_*	n_cases_	HR (95% CI)	*p _trend_*	n_cases_	HR (95% CI)	*p _trend_*
BMI (kg/m^2^ ^a^									
Continuous per kg/m^2^		0.98 (0.93‐1.04)	0.520		0.97 (0.90‐1.06)	0.507		0.97 (0.93‐1.00)	0.079
Categories									
Underweight (<18.5)	1	0.99 (0.14‐7.15)		1	1.97 (0.27‐14)		6	0.89 (0.40‐2.02)	
Normal weight (18.5‐24.9)	54	1.00		165	1.00		160	1.00	
Overweight (25.0‐29.9)	30	1.02 (0.65‐1.60)		210	0.26 (0.09‐0.73)		42	0.83 (0.59‐1.16)	
Obese (≥30.0)	8	0.69 (0.32‐1.46)		18	1.37 (0.59‐3.14)		7	0.46 (0.22‐0.99)	
Physical activity^b^									
Continuous per category		0.65 (0.45‐0.94)	0.022		0.82 (0.52‐1.31)	0.393		1.16 (1.00‐1.34)	0.051
Categories									
Sedentary	26	1.00		11	1.00		41	1.00	
Moderately active	60	0.63 (0.39‐1.00)		30	0.71 (0.35‐1.42)		154	1.41 (0.99‐1.99)	
Active	7	0.47 (0.20‐1.08)		6	0.80 (0.29‐2.18)		18	1.21 (0.69‐2.11)	
Systolic blood pressure (mm Hg)^c^									
Continuous per 10 mmHg		1.03 (0.92‐1.15)	0.590		1.01 (0.85‐1.22)	0.880		1.03 (0.94‐1.12)	0.564
Categories									
Normal (<130)	43	1.00		27	1.00		132	1.00	
High normal (130‐139)	26	1.45 (0.89‐2.38)		11	1.31 (0.64‐2.66)		41	1.05 (0.74‐1.49)	
Hypertension (≥140)	24	0.88 (0.51‐1.51)		9	1.02 (0.43‐2.41)		42	1.15 (0.81‐1.65)	
Diastolic bloodpressure (mm Hg)^c^									
Continuous per 10 mm Hg		0.86 (0.70‐1.05)	0.143		1.10 (0.83‐1.45)	0.511		1.00 (0.88‐1.14)	0.977
Categories									
Normal (<85)	67	1.00		30	1.00		167	1.00	
High normal (85‐89)	15	1.21 (0.69‐2.13)		8	1.99 (0.90‐4.37)		17	0.75 (0.45‐1.23)	
Hypertension (≥90)	11	0.53 (0.27‐1.02)		9	2.20 (1.01‐4.37)		31	1.06 (0.72‐1.57)	
Triglycerides (mmol/L)^c^									
Continuous per mmol/L		0.94 (0.70‐1.25)	0.662		0.98 (0.65‐1.47)	0.912		0.91 (0.75‐1.11)	0.362
Categories									
Normal (<1.7)	68	1.00		40	1.00		166	1.00	
Borderline high (1.7‐2.2)	12	1.19 (0.63‐2.22)		4	0.74 (0.26‐2.11)		34	1.15 (0.79‐1.68)	
High (≥ 2.3)	13	1.55 (0.82‐2.91)		3	0.77 (0.23‐0.59)		15	0.68 (0.39‐1.17)	
Total cholesterol (mmol/L)^c^									
Continuous per mmol/L		0.93 (0.78‐1.13)	0.480		1.16 (0.92‐1.46)	0.213		1.05 (0.94‐1.18)	0.392
Categories									
Normal (<5.2)	27	1.00		16	1.00		53	1.00	
Borderline high (5.2‐6.1)	30	0.83 (0.49‐1.40)		17	0.95 (0.48‐1.90)		72	1.06 (0.74‐1.51)	
High (≥6.2)	34	0.83 (0.48‐1.44)		14	0.94 (0.44‐2.02)		87	1.31 (0.92‐1.87)	

Note

Cox proportional hazard regression models were adjusted for:

^a^ Adjusted for age as the underlying time scale, physical activity, education, and high risk occupation

^b^ Adjusted for age as the underlying time scale, BMI, education, and high risk occupation

^c^ Adjusted for age as the underlying time scale, BMI, physical activity, education, and high risk occupation

^d^ Former and current smokers were additionally adjusted for pack years.

Using the risk score, integrating information across the lifestyle associated factors, we found a positive association with BC risk in men (*p*_trend_ = 0.043) (Table 5). When stratifying by smoking status, a positive association was significant for former smokers only (*p*_trend_ = 0.045) and never smokers with a risk score of 3 had elevated BC risk (HR 1.70, 95% CI 1.06‐2.71) compared to risk score 0. In women, there was no association between risk score and BC risk (Supplementary, Table S4).

**TABLE 5:** Hazard ratio (HR) with 95% confidence interval (CI) of bladder cancer risk among men according to a lifestyle associated risk score

Risk score	All men			Never Smoker			Former smoker ^a^			Current smoker ^a^		
	n_cases_	HR (95% CI)	*p_trend_*	n_cases_	HR (95% CI)	*p_trend_*	n_cases_	HR (95% CI)	*p_trend_*	n_cases_	HR (95% CI)	*p_trend_*
0	235	1 (base)	0.043	30	1 (base)	0.270	46	1 (base)	0.045	155	1 (base)	0.600
1	427	1.04 (0.88‐1.22)		56	1.29 (0.83‐2.01)		91	1.04 (0.73‐1.49)		271	0.95 (0.78‐1.16)	
2	424	1.05 (0.89‐1.23)		54	1.36 (0.87‐2.13)		121	1.34 (0.95‐1.89)		247	0.88 (0.72‐1.08)	
3	319	1.11 (0.94‐1.32)		45	1.70 (1.06‐2.71)		76	1.12 (0.77‐1.62)		197	0.99 (0.80‐1.22)	
4 or 5	200	1.18 (0.98‐1.43)		14	0.99 (0.52‐1.87)		57	1.45 (0.97‐2.14)		128	1.05 (0.83‐1.33)	

Note

Cox proportional hazard regression models were adjusted for age as the underlying time scale, education, and high risk occupation.

^a^ Former and current smokers were additionally adjusted for pack years.

Analyses according to established risk factors for BC showed increased risk of BC in current smokers compared to never smokers, both for men (HR 2.95, 95% CI 2.53‐3.45) and women (HR 2.70, 95% CI 2.10‐3.48) (Supplementary Table**** S5). In men, increased risk was also seen for former smokers (HR 1.70, 95% CI 1.43‐2.02) compared to never smokers. Furthermore, total smoking exposure was associated with increased risk, shown by a linear increase in risk with increasing pack years, in both men (HR 1.02, 95% CI 1.02‐1.03) and women (HR 1.04, 95% CI 1.02‐1.06). High risk occupations were associated with increased BC risk in men (HR 1.19, 95% CI 1.07‐1.33). Use of blood pressure medication was not associated with BC risk (data not shown).

Regarding tumor invasiveness, looking at NMIBC and MIBC as separate BC outcomes, results and conclusions did not differ from the results presented in Table 2 (data not shown).

## 4 DISCUSSION

In this prospective cohort study with nearly 2000 BC cases, we found that elevated blood pressure, both DBP and SBP, was positively associated with BC risk in men. Stratification by smoking revealed that the association between DBP and BC was most pronounced among never smokers. A risk score, integrating information across the studied lifestyle associated factors was positively associated with BC risk in men. In women, physical activity was associated with decreased BC risk, but only among never smokers. Overall, associations between lifestyle associated factors and BC risk were most evident in never smokers.

In the present study, elevated blood pressure was the strongest risk factor among men. The Me‐Can project is the largest study showing associations between hypertension and BC risk in men after adjustment for pack years.^33^, ^50^ In our study cohort, which is a subpopulation of the Me‐Can study, we found similar results, even after adjusting for other potential confounders, such as occupational exposure and physical activity. Other studies have shown associations between hypertension and bladder cancer risk, but the literature is not consistent.^27^, ^51^, ^52^, ^53^ However, the majority of these studies have limitations such as self‐reported data on hypertension status, or insufficient information on smoking habits.

There is increasing evidence from epidemiological studies for an association between hypertension and cancer risk, however the underlying biological mechanism remains unclear.^28^, ^54^ One of the challenges is that hypertension is also linked to well‐established risk factors for cancer such as smoking and obesity.^55^, ^56^ Thus, we have adjusted for such potential confounding and stratified the analysis by smoking status revealing an even stronger association in never smokers. However, we cannot exclude the possibility of residual confounding from factors not adequately captured (eg central obesity, which is not necessarily reflected by BMI).^28^, ^57^

Previous studies have had difficulties in separating the effect of hypertension from the intake of antihypertensive drugs on the risk of cancer.^58^ We had self‐reported information about use of antihypertensive drugs, and found no associations between antihypertensive treatment and BC risk. In addition, the observed dose‐response relationship, showing a positive relation between increasing DBP and BC risk in men, strengthens the hypothesis that hypertension is the risk factor, other than factors related to hypertension.

Several meta‐analyses have found positive associations between high BMI and BC risk, although few single studies have managed to show significant associations.^22^, ^59^, ^60^ A majority of these studies lacked information about smoking intensity and duration. We found no positive associations, neither for overweight nor obesity compared to normal BMI, in either sexes, when adjusting for both smoking status and pack years. However, after stratification by smoking status, we found that male former smokers with overweight had an increased risk of BC. This finding is in line with a study by Roswall et al who found an association between overweight and BC risk for male former smokers.^19^ This could be a result of residual confounding by smoking, or it may be connected to smoking cessation being associated with weight gain, and that former smokers that gain weight after quitting might have been the heaviest smokers.^30^ On the contrary, in women, a decreased risk was found in former smokers with overweight and in current smokers that were obese. A similar association was seen by Roswall et al, who found a nonsignificant inverse association between BMI and BC risk in women. BMI has also been inversely associated with risk of lung cancer, and this relationship is primarily restricted to smokers.^61^ Given that female smokers were on average leaner than never smokers, the inverse relationship between BMI and BC could probably be explained by residual confounding by smoking.

We found no statistical significant associations between serum levels of triglycerides and total cholesterol risk of BC. The Me‐Can study found a positive association between triglycerides and BC incidence among men, but not for cholesterol,^33^ while other studies have reported no associations, ^49^, ^62^ as in the current study. The serum samples used were collected without any fasting restrictions. Thus, variations in food‐intake close to blood‐draw have most likely influenced the triglyceride level in the samples and could have led to nondifferential misclassification of measured levels and bias the findings toward the null.

In men, high physical activity tended to reduce the risk of BC, although the finding was not statistically significant (*p*_rend_ = 0.07). In women, high physical activity was associated with reduced risk among never smokers, with a tendency also for former smokers. For current smokers, however, there was a tendency of an increased risk. Previous studies have shown that physical activity may reduce BC risk, however, the results have not been consistent.^18^, ^23^, ^63^ Most studies, including ours, are based on self‐reported data that might lead to nondifferential misclassifications of physical activity, which may bias a potential association with bladder cancer toward the null. A recent study, based on measured cardiorespiratory fitness, found that high cardiorespiratory fitness was associated with a 60% decreased risk of BC, which might indicate a role of physical activity in the etiology of BC.^64^

We also found a positive association between a lifestyle associated risk score and BC risk in men, whereas no association was observed among women. This is in line, with other studies reporting that metabolic syndrome, which is a combination of high BMI, dyslipidemia, hypertension, and impaired glucose levels, is associated with increased risk of BC in men, but not in women.^17^, ^50^ Increasing evidence in the literature also support that metabolic disorders associated with BMI could be a stronger predictor of cancer than BMI per se,^26^ which is in agreement with our findings. Stratification by smoking revealed that the association between the combined risk score and BC risk was most pronounced among former and never smokers, which could indicate that smoking is such a strong risk factor that it diminishes the effects of the lifestyle associated factors on BC risk in current smokers.

Our results confirmed that smoking is by far the strongest lifestyle associated risk factor for BC with a threefold increased BC risk for smokers, in line with previous studies.^65^ This introduces a challenge when studying other lifestyle associated factors that also are related with smoking. For instance, individuals who smoke are generally less physically active, have lower body weight, and are more likely to have higher levels of triglycerides and cholesterol,^30^, ^31^ which is also confirmed in our cohort. In light of the complex relation between smoking status and the factors in focus, the associations found in never smokers might be the most reliable results, not confounded by smoking. Our data also confirmed that high risk occupation is a strong risk factor for BC in men, even after adjustment for smoking status and pack years. In women, no such association was found, probably due to few women with high risk occupations (3%). Few other studies of the relation between lifestyle and BC have been able to adjust for occupational exposure, which is an important factor to take into account, being associated with both lifestyle and BC.^10^

Overall, the lack of significant findings in women could partly be explained by a lower power to detect associations as women have a lower prevalence of both lifestyle associated risk factors and BC incidence. Furthermore, there might be sex‐specific biological differences involved, including body fat distribution and hormonal regulation of bodyweight that could interplay differently in relation to metabolic health and cancer.^66^, ^67^ In addition, sex‐hormones have been suggested to influence bladder carcinogenesis differently, testosterone has been suggested to promote cancer, whereas estrogen may protect BC development.^68^

Our results did not differ with tumor aggressiveness, comparing associations in NMIBC and MIBC, respectively. This is in line with other studies,^19^, ^33^ although Teleka et al found some differences in associations when investigating the relationship between metabolic factors and BC by tumor invasiveness, they concluded, however, that the associations were generally not different.^33^

Strengths of our study include the prospective design, the long follow‐up period, and the inclusion of almost 2000 primary BC cases, with high quality and complete cancer data from a population based cancer registry. The prediagnostic measurements of BMI and blood pressure were performed by trained personnel according to a standardized protocol. Moreover, we had detailed information about smoking, including information about smoking intensity and duration that allowed us to create a pack years variable. Of note, the smoking variables were self‐reported, which has been shown to underreport true smoking.^69^ In addition, we had information on high risk occupations that is the second most established risk factor.

There were also some limitations in our study. All measurements were performed at baseline, at the time where the majority of the study population was around 40 years. As the mean age of BC incidence was around 60 years, measurements of the cohort members were generally performed long time prior to the cancer diagnosis. However, it is shown that lifestyle habits established mid‐life as a good predictor of late‐life outcomes including cancer development.^70^ Moreover, most of the lifestyle associated factors in focus are collected in the 1970s and 1980s, reflecting a different lifestyle compared to the western world today.^71^ Many of our cohort members were heavy smokers, and far less were overweight and obese compared to the current general population. Thus, smoking may have overshadowed some of the effects from obesity and related metabolic disorders.

In conclusion, hypertension, seemed to be an independent risk factor for BC in men. The association was stronger for DBP than SBP, and stratification by smoking revealed that the effect was most pronounced among never smokers, supporting that the association is independent of smoking. In addition, a combined risk score of lifestyle associated factors modestly increased BC risk in men. This result seemed to be most prominent in never smokers and former smokers. In women, physical activity seems to protect against BC, but only among never smokers. Overall, associations between lifestyle associated factors and BC risk were more evident among never smokers and former smokers, suggesting that smoking dominates the effects in current smokers. Even though we had robust information on smoking habits, we cannot rule out that confounding by smoking might persist. Future studies of lifestyle associated factors and BC risk should probably be performed among never smokers, to secure better confounding control.

## CONFLICT OF INTEREST

The authors declare no competing interests.

## Supporting information

Table S1‐S5

## Data Availability

The data are available as presented in the paper. According to Norwegian legislation, our approvals to use the data for the current study do not allow us to distribute or make the data directly available to other parties.
