# Supplementary material for: Lifestyle associated factors and risk of urinary bladder cancer: A prospective cohort study from Norway
Source: Cancer Med. 2020 Apr 21;9(12):4420–32. doi: 10.1002/cam4.3060 (PMC7300409; doi:10.1002/cam4.3060)
Supplement: Supplementary file 1 — Table S1‐S5 [file CAM4-9-4420-s001.docx]

**Supplementary tables S1-S6**

**Table S1** List of occupations classified as high-risk occupations, based on working titles associated with bladder cancer risk, and chemical exposures related to bladder cancer risk, such as aromatic amines, polycyclic hydrocarbons, aromatic hydrocarbons, diesel

| **Working title** | **Men (N/cases)** | **Women (N,cases)** |
| --- | --- | --- |
| **Chemists and laboratory assistance** | 694/8 | 953/2 |
| Chemists |  |  |
| Laboratory assistants |  |  |
| **Miners and quarrymen** | 1,160/12 | 21/0 |
| Miners, shot firers etc. |  |  |
| Miners and quarrymen |  |  |
| **Railway and motor transport work** | 13,017/184 | 825/1 |
| Retailers and leaseholders |  |  |
| Railway engine drivers, steam engine firemen |  |  |
| Bus drivers |  |  |
| Tram and metro drivers |  |  |
| Taxi drivers |  |  |
| Van and lorry drivers |  |  |
| Other motor vehicle drivers |  |  |
| Harbour masters |  |  |
| Air traffic controllers |  |  |
| Railway traffic supervisors |  |  |
| Road transport supervisors |  |  |
| **Upholsters and shoe workers** | 289/ 9 | 348/0 |
| Upholsterers |  |  |
| Leather cutters for footwear |  |  |
| Shoe sewers |  |  |
| Lasters and sole fitters etc. |  |  |
| Footwear workers, nec |  |  |
| **Metal Workers** | 20,505/222 | 565/5 |
| Metal smelting furnacemen |  |  |
| Heat treaters, hardeners, temperers etc. |  |  |
| Hot-rolling metal workers |  |  |
| Cold-rolling metal workers |  |  |
| Wire and pipe drawers |  |  |
| Smiths |  |  |
| Foundry workers |  |  |
| Occupations in smelting, metallurgical and foundry work |  |  |
| Turners, toolmakers and machine-tool setters |  |  |
| Fitter-assemblers etc |  |  |
| Machine and engine mechanics |  |  |
| Sheet metal workers |  |  |
| Plumbers |  |  |
| Welders and flame cutters |  |  |
| Sheet metal formers, steel constructions |  |  |
| Metal plating and coating work |  |  |
| Assemblers and other machine and metalware occupations |  |  |
| **Painters and printers** | 3,083/47 | 383/0 |
| Building painters etc. |  |  |
| Lacqurers |  |  |
| Other painters, lacquerers and floor layers |  |  |
| Lithographers |  |  |
| Printers |  |  |
| Bookbinders |  |  |
| Occupation in graphics, nec |  |  |
| **Chemical process workers** | 850/14 | 132/1 |
| Distillers |  |  |
| Cookers and furnacemen (chemical processes) |  |  |
| Other occupations in the chemical industry |  |  |
| **Rubber and plastic industry** | 1,454/16 | 314/2 |
| Concrete-mixer operators and cast concrete product workers |  |  |
| Rubber products workers |  |  |
| Plastic product workers |  |  |
| **Engine operators** | 4,706/60 | 50/0 |
| Stationary engine and machinery operators (not on vessels) |  |  |
| Crane operators etc. |  |  |
| Forklift operators etc. |  |  |
| Greasers etc. |  |  |
| Engine-room crew |  |  |
| Motormen |  |  |
| Steam engine firemen |  |  |
| Other engine workers |  |  |
| **Hairdressers** | 293/ 3 | 1,319/4 |
| **Laundry workers** | 128/ 0 | 536/2 |

**Table S2** Characteristics of study population among men, stratified by smoking status

|  | **Never smoker** | **Former smoker** | **Current smoker** |
| --- | --- | --- | --- |
| **Bladder cancer cases (n, % of cases)** | 201 | 395 | 1006 |
| **BMI, kg/m^2^** |  |  |  |
| Mean (SD) | 25.2 (3.1) | 25.7 (3.1) | 24.9 (3.2) |
| Categories n (%) |  |  |  |
| Underweight (< 18.5) | 139 (0.35) | 93 (0.2) | 444 (0.7) |
| Normal weight (18.5-24.9) | 20,542 (51) | 18,016 (44) | 37,982 (55) |
| Overweight (25.0-29.9) | 16,460 (41) | 19,297 (47) | 25,901 (38) |
| Obese (>30.0) | 2,783 (7) | 3,313 (8) | 4,239 (6) |
| **Physical activity, n (%)** |  |  |  |
| Inactive | 6,084 (15) | 6,646 (16) | 16,565 (24) |
| Moderately active | 19,638 (49) | 21,878 (54) | 36,787 (54) |
| Active | 13,988 (35) | 12,030 (29) | 15,095 (22) |
| **Sytosolic bloodpressure, mmHg** |  |  |  |
| Mean (SD) | 136 (15) | 137 (16) | 135 (16) |
| *Category, n (%)* |  |  |  |
| Normal (<130) | 14,081 (35) | 13,878 (34) | 25,698 (37) |
| High normal (130-139) | 11,151 (28) | 11,143 (27) | 18,883 (27) |
| Hypertension (≥140) | 14,743 (37) | 15,765 (39) | 24,124 (35) |
| **Diastolic blood pressure, mmHg** |  |  |  |
| *Mean (SD)* | 83 (11) | 84 (11) | 83 (11) |
| *Category, n (%)* |  |  |  |
| Normal (<85) | 24,116 (60) | 22,859 (56) | 41,118 (60) |
| High normal (85-89) | 5,856 (15) | 6,163 (15) | 10,030 (15) |
| Hypertension (≥90) | 10,002 (25) | 11,765 (29) | 17,554 (26) |
| **Triglycerides, mmol/L** |  |  |  |
| Mean (SD) | 1.9 (1.2) | 2.1 (1.3) | 2.1 (1.4) |
| *Category, n (%)* |  |  |  |
| Normal (<1.7) | 21,156 (53) | 19,834 (49) | 31,574 (46) |
| Borderline high (1.7-2.2) | 8,132 (20) | 8,689 (21) | 15,224 (22) |
| High (≥ 2.3) | 10,731 (27) | 12,282 (30) | 21,926 (32) |
| **Cholesterol, mean mmol/L** |  |  |  |
| Mean (SD) | 5.8 (1.1) | 6.1 (1.2) | 6.2 (1.2) |
| *Category, n (%)* |  |  |  |
| Normal (<5.2) | 11,748 (29) | 9,211 (23) | 13,891 (20) |
| Borderline high (5.2-6.1) | 15,030 (38) | 14,926 (37) | 23,667 (34) |
| High (≥ 6.2) | 13,244(33) | 16,675 (41) | 31,173 (45) |
| **High risk occupation, n (%)** |  |  |  |
| No | 29,421 (74) | 27,667 (68) | 42,436 (62) |
| Yes | 9,083 (23) | 12,530 (31) | 24,615 (36) |
| **Education, n (%)** |  |  |  |
| None | 257 (0.6) | 68 (0.17) | 165 (0.2) |
| Compulsory | 7,951 (20) | 11,317 (28) | 26,369(38) |
| Upper sec. | 19,486 (49) | 20,668 (51) | 33,450 (49) |
| College/University | 12,221 (31) | 8,637 (21) | 8,423 (12) |

**Table S3,** Characteristics of study population among women, stratified by smoking status

|  | **Never smoker** | **Former smoker** | **Current smoker** |
| --- | --- | --- | --- |
| **Bladder cancer cases (n, % of cases)** | 93 | 47 | 215 |
| **BMI, kg/m^2^** |  |  |  |
| Mean (SD) | 24.7 (4.1) | 24.5 (3.9) | 23.6 (3.8) |
| BMI |  |  |  |
| Underweight (< 18.5) | 725 (1) | 274 (1) | 1,776 (3) |
| Normal weight (18.5-24.9) | 33,597 (61) | 16,544 (64) | 38,376 (69) |
| Overweight (25.0-29.9) | 14,974 (27) | 6,751 (26) | 11,788 (21) |
| Obese (>30.0) | 5,580 (10) | 2,197 (9) | 3,656 (7) |
| **Physical activity, n (%)** |  |  |  |
| Inactive | 10,057 (18) | 4,629 (18) | 13,538 (24) |
| Moderately active | 38,433 (70) | 17,815 (69) | 36,930 (66) |
| Active | 6,155 (11) | 3,140 (12) | 4,895 (9) |
| **Sytosolic bloodpressure, mmHg** |  |  |  |
| Mean (SD) | 130 (18) | 127 (16) | 127(16) |
| *Category, n (%)* |  |  |  |
| Normal (<130) | 30,511 (55) | 16,269 (63) | 35,276 (63) |
| High normal (130-139) | 10,981 (20) | 4,795 (19) | 10,526 (19) |
| Hypertension (≥140) | 13,532 (25) | 4,752 (18) | 9,877 (18) |
| **Diastolic blood pressure, mmHg** |  |  |  |
| *Mean (SD)* | 79 (11) | 78 (11) | 78 (11) |
| *Category, n (%)* |  |  |  |
| Normal (<85) | 39,659 (72) | 19,873 (77) | 42,093 (76) |
| High normal (85-89) | 6,247 (11) | 2,599 (10) | 5,958 (11) |
| Hypertension (≥90) | 9,117 (17) | 3,345 (13) | 7,629 (14) |
| **Triglycerides, mmol/L** |  |  |  |
| Mean (SD) | 1.3 (0.8) | 1.3 (0.8) | 1.4 (0.9) |
| *Category, n (%)* |  |  |  |
| Normal (<1.7) | 43,972 (80) | 21,081 (82) | 41,924 (75) |
| Borderline high (1.7-2.2) | 6,130 (11) | 2674 (10) | 7,621 (14) |
| High (≥ 2.3) | 4,934 (9) | 2,068 (8) | 6,148 (11) |
| **Cholesterol, mean mmol/L** |  |  |  |
| Mean (SD) | 5.7 (1.1) | 5.6 (1.1) | 5.8 (1.4) |
| *Category, n (%)* |  |  |  |
| Normal (<5.2) | 18,594 (34) | 9,736 (38) | 16,534 (30) |
| Borderline high (5.2-6.1) | 20,529 (37) | 9,691 (38) | 20,178 (37) |
| High (≥ 6.2) | 15,913(29) | 6,399 (25) | 18,435 (33) |
| **High risk occupation, n (%)** |  |  |  |
| No | 43,289 (79) | 20,557 (80) | 42,543 (76) |
| Yes | 1672 (3) | 1,073 (4) | 2,598 (5) |
| **Education, n (%)** |  |  |  |
| None | 283 (0.5) | 38 (0.15) | 95 (0.17) |
| Compulsory | 15,447 (28) | 7,409 (29) | 23,627 (42) |
| Upper secondary | 27,741 (50) | 13,398 (52) | 26,978 (48) |
| College/University | 11,431 (21) | 4,899 (19) | 4,798 (9) |
|  |  |  |  |

**Table S4** Hazard ratio (HR) with 95% confidence interval (CI) of bladder cancer risk among women according to a lifestyle associated risk score

| **Risk score** |  |  | **All women** |  |
| --- | --- | --- | --- | --- |
|  |  | n_cases_ | HR (95% CI) | *p _trend_* |
| **0** |  | 108 | 1 (base) |  |
| **1** |  | 125 | **1.08** (0.84-1.40) |  |
| **2** |  | 75 | **1.01** (0.74-1.36) |  |
| **3** |  | 36 | **0.92** (0.63-1.36) |  |
| **4 or 5** |  | 13 | **0.80** (0.44-1.43) |  |
|  |  |  |  | 0.463 |

Cox proportional hazard regression models were adjusted for age as the underlying time scale, smoking status and packyears education and high risk occupation

**Table S5** Hazard ratio (HR) with 95% confidence interval (CI) of bladder cancer according to smoking status, smoking status and packyears, high risk occupation and education

|  | **Men** | | | **Women** | | | |
| --- | --- | --- | --- | --- | --- | --- | --- |
|  | n_cases_ | **HR (95% CI)** | ***p*** | n_cases_ | **HR (95% CI)** | ***p*** | |
| **Smoking status^†^** |  |  |  |  |  |  | |
| Never (base) | 201 | 1.00 |  | 93 | 1.00 |  | |
| Former | 395 | 1.70 (1.43, 2.02) | 0,000 | 47 | 1.25 (0.88, 1.77) | 0,220 | |
| Current | 1006 | 2.95 (2.53, 3.45) | 0,000 | 215 | 2.70 (2.10, 3.48) | 0,000 | |
| **Smoking status and packyears^†^** |  |  |  |  |  |  | |
| Never | 201 | 1.00 |  | 93 | 1.00 |  | |
| Former | 395 | 1.70 (1.43, 2.02) | 0,000 | 47 | 1.25 (0.88,1.78) | 0,201 | |
| Current, packyears 5Q |  |  |  |  |  |  | |
| Q1 | 79 | 1.88 (1.45, 2.44) | 0,000 | 52 | 2.30 (1.63-3.24) | 0,000 | |
| Q2 | 172 | 2.81 (2.29, 3.46) | 0,000 | 44 | 2.24 (1.55, 3.22) | 0,000 | |
| Q3 | 184 | 2.86 (2.33, 3.50) | 0,000 | 49 | 3.17 (2.22, 4.52) | 0,000 | |
| Q4 | 189 | 3.19 (2.61, 3.90) | 0,000 | 24 | 2.63 (1.66, 4.15) | 0,000 | |
| Q5 | 286 | 3.64 (3.02, 4.38) | 0,000 | 33 | 4.41 (2.93, 6.62) | 0,000 | |
| Packyears, continuous per packyear |  | 1.02 (1.02,1.03) | 0,000 |  | 1.04 (1.02, 1.06) | 0,000 | |
| **High risk occupation ^‡^** |  |  |  |  |  |  | |
| No | 1023 | 1.00 |  | 279 | 1.00 |  | |
| Yes | 575 | 1.19 (1.07, 1.33) | 0,001 | 17 | 1.13 (0.69, 1.84) | 0,630 | |
| **Education ^§^** |  |  |  |  |  |  | |
| None | 2 | 0.46 (0.11, 1.85) | 0.274 | 1 | 1.17 (0.16, 8.42) | 0,874 | |
| Compulsory | 603 | 1.00 |  | 149 | 1.00 |  | |
| Upper sec. | 750 | 0.93 (0.83, 1.03) | 0.172 | 163 | 0.91 (0.73, 1.15) | 0,443 | |
| College/University | 257 | 0.95 (0.81, 1.10) | 0.481 | 46 | 0.99 (0.70, 1.40) | 0,951 | |
|  |  |  |  |  |  |  | |
| Cox proportional hazard regression models were adjusted for: | | | | | | |  |
| ^†^ Adjusted for age as the underlying time scale, BMI, physical activity, education and high risk occupation | | | | | | |  |
| ^‡^ Adjusted for age as the underlying time scale, BMI, physical activity, smoking status and packyears, education and high risk occupation | | | | | | |  |
| ^§^ Adjusted for age as the underlying time scale, BMI, physical activity, smoking status and packyears, and high risk occupation | | | | | | |  |
